# The Epidemiology and Pathogenesis of Kawasaki Disease

**DOI:** 10.3389/fped.2018.00374

**Published:** 2018-12-11

**Authors:** Anne H. Rowley, Stanford T. Shulman

**Affiliations:** Department of Pediatrics, Northwestern University Feinberg School of Medicine, The Ann & Robert H. Lurie Children's Hospital of Chicago, Chicago, IL, United States

**Keywords:** kawasaki, pathogenesis, etiology, pediatric, coronary

## Abstract

Epidemiologic and clinical features of Kawasaki Disease (KD) strongly support an infectious etiology. KD is worldwide, most prominently in Japan, Korea, and Taiwan, reflecting increased genetic susceptibility among Asian populations. In Hawaii, KD rates are 20-fold higher in Japanese ethnics than in Caucasians, intermediate in other ethnicities. The age distribution of KD, highest in children < 2 yo, lower in those < 6 months, is compatible with infection by a ubiquitous agent resulting in increasing immunity with age and with transplacental immunity, as with some classic viruses. The primarily winter-spring KD seasonality and well-documented Japanese epidemics with wave-like spread also support an infectious trigger. We hypothesize KD pathogenesis involves an RNA virus that usually causes asymptomatic infection but KD in a subset of genetically predisposed children. CD8 T cells, oligoclonal IgA, and upregulation of cytotoxic T cell and interferon pathway genes in the coronaries in fatal KD also support a viral etiology. Cytoplasmic inclusion bodies in ciliated bronchial epithelium identified by monoclonal antibodies made from oligoclonal IgA heavy chains also supports a viral etiology. Recent availability of “second generation” antibodies from KD peripheral blood plasmablasts may identify a specific viral antigen. Thus, we propose an unidentified (“new”) RNA virus infects bronchial epithelium usually causing asymptomatic infection but KD in a subset of genetically predisposed children. The agent persists in inclusion bodies, with intermittent respiratory shedding, entering the bloodstream via macrophages targeting coronaries. Antigen-specific IgA plasma cells and CD8 T cells respond but coronaries can be damaged. IVIG may include antibody against the agent. Post infection, 97–99% of KD patients are immune to the agent, protected against recurrence. The agent can spread either from those with asymptomatic primary infection in winter-spring or from a previously infected contact who intermittently sheds the agent.

## Epidemiology

Both epidemiologic and clinical features of Kawasaki Disease (KD) strongly support an Infectious etiology. The clinical features of KD including fever, rash, mucosal changes, conjunctival erythema, and cervical lymphadenopathy are all compatible with an infectious illness, and many common (predominantly viral) infections by necessity are included in the differential diagnosis of KD.

Kawasaki Disease (KD) is a worldwide illness, with varying incidence rates that primarily reflect the racial composition of the populations of various countries. The highest incidence of KD is in Japan, and this has steadily increased with an annual rate of 308.0 per 100,000 children under 5 years reported in 2014 (1). In Japan one in 65 children develops KD by age 5 years. The second highest reported rate was 199.7 per 100,000 < 5 years old in 2014 in South Korea (2), while Taiwan has the third highest rate, 82.8 per 100,000 < 5 years old in 2010 (3). In countries with predominantly non-Asian populations, the usual annual rate is 10–20 per 100,000 <5 years old (4).

More than 15,979 cases of KD were reported in Japan in 2015, with local clusters occurring commonly, unlike the nationwide epidemics that occurred in 1979, 1982, and 1985–86 (1, 5). In those epidemics, there appeared to be wave-like spread from one prefecture to an adjacent one, a pattern very similar to the spread of specific viral illnesses like measles, for example, in Japan, thus strongly supporting an infectious etiology of KD. In Hawaii, with its complex multi-racial and multi-ethnic population, the overall annual KD incidence is about 50.4/100,000 < 5 y/o; for Japanese ethnic children in Hawaii, the rate is about 210.5 and for Caucasians about 13.7, with intermediate rates for children of native Hawaiian, Chinese, Filipino and other Asian ancestries (6). The very striking differences in ethnic-specific rates are indicative of a very strong genetic basis of susceptibility.

The ratio of male: female KD patients approximates 1.5:1 in virtually all countries (1, 4), and severe cardiac complications of KD are even more significantly overrepresented in males. The basis of the male preponderance is unclear but similar to that observed in many infectious diseases.

Kawasaki Disease (KD) has a striking age distribution, with almost 100% of cases occurring in children, 80% in children <5 years old, and 50% in those <2 years old. In a recent Japanese survey 0.7% of cases were ≥ 10 years old (1). The age-incidence curve of KD may help to elucidate risk factors and appears compatible with a ubiquitous highly transmissible infectious agent, and is similar to that seen with respiratory syncytial virus (RSV), for example. The peak age of KD is approximately 10–11 months of life, with a relatively low incidence in the first 6 months, suggesting both the possibility of transplacental immunity as seen in many classic infectious illnesses, as well as progressively increasing degrees of immunity to the KD agent throughout childhood.

The seasonality of KD, with winter peaks in Japan and winter-spring predominance in the US and many other temperate areas, is highly suggestive of a viral (probably respiratory viral) etiology (4, 7). Some reports have suggested summer and winter peaks (Beijing and Shanghai), or spring peaks (Sichuan and Hong Kong), while no clear seasonality has been seen in Hawaii (6), and winter predominance was reported from at least some Southern Hemisphere countries. Despite the observed seasonality, in most areas sporadic cases are recognized throughout the year, contrasting somewhat with the usual patterns commonly seen with many highly transmissible respiratory viral illnesses. Recurrent KD is defined as a new illness that meets KD criteria beginning at least 3-months and usually within 2 years after an initial episode of KD, when levels of inflammatory markers have completely normalized. Recurrence occurs in about 1% or fewer of all KD patients, and in up to 3% of those of Asian ethnicity (8).

During an outbreak of KD on Mikayo Island, Japan, in 1980–1981 (a fairly isolated population of ~80,000 at that time), 9 KD cases were diagnosed in a 1 month period, and 4 of the cases had close geographic and social contacts, supporting the possibility of direct person-to-person transmission of a KD etiologic agent (9). While there is limited other direct evidence to indicate that KD can be transmitted from person to person, for example in a daycare setting, much circumstantial evidence supports an infectious etiology with genetically susceptible individuals manifesting the clinical features of KD and others having trivial or no symptoms. Simultaneous or sequential cases in siblings, twins, or other contacts are reported, especially during Japanese outbreaks (10). In Japan, secondary sibling cases occur at rates substantially higher than the general childhood population. Sibling cases are reported more frequently in twins than in non-twins, suggesting both genetic susceptibility and person to person transmission. Japanese family data suggest that sibling cases tend to cluster either on the same day as the index case or 7 days later (11).

History of increased frequency of antecedent respiratory illnesses in KD compared to controls was documented in the 1980's in several outbreak investigations (12, 13). Together with the epidemiologic features noted above, the clinical features characteristic of KD also strongly suggest that an infectious agent, perhaps one that has not yet been identified as a human pathogen, is etiologically related to KD.

## Pathogenesis

The epidemiologic features of KD described above strongly support infection with a ubiquitous agent that usually results in asymptomatic infection, but causes KD in a small subset of genetically predisposed children. The occurrence of epidemics and geographic wave-like spread of KD during epidemics supports a presently unknown single agent or closely related group of agents as the etiology. The failure of KD patients to respond to antibiotic therapy makes a viral etiology more likely than a bacterial cause. Moreover, the prevalence of CD8 T cells in the inflammatory infiltrate and the upregulation of cytotoxic T cell and interferon pathway genes in the coronary arteries of children who have died of KD are very suggestive of a viral etiology (14, 15).

We discovered an oligoclonal IgA response in the coronary arteries of children who died from KD, and we made “first generation” KD synthetic antibodies using oligoclonal IgA heavy chains with random light chains (16–19). These “first generation” antibodies detected antigen residing in ciliated bronchial epithelium in KD lung and in a subset of macrophages in KD but not in infant control tissues by immunohistochemistry; the antigen in lung localized to intracytoplasmic inclusion bodies that were identified using stains for protein and for RNA (20–22). The inclusion bodies were identified in children from the US and Japan using a single monoclonal antibody, strongly suggesting a single infectious agent as the cause (20, 22, 23). The inclusion bodies could also be identified in some KD children who died as late as months to years after onset (21). Further investigations of acute phase KD lung samples showed upregulation of interferon pathway genes and virus-like particles in close proximity to the inclusion bodies by transmission electron microscopy (23). However, these antibodies did not identify the specific antigen by Western blot and immunoprecipitation assays, likely because of a lack of cognate heavy and light chain partners in these “first generation” antibodies. This problem has been recently overcome by preparing “second generation” antibodies from acute KD peripheral blood plasmablasts, which include cognate light and heavy chain partners and show great promise in identifying specific antigen (24).

One theory presently favored by some is that KD can result from infection with any of a wide range of infectious agents in a genetically predisposed host, and some investigators propose an immune defect in KD children. We believe that these theories fail to explain epidemiologic findings in KD. If multiple agents can cause KD, epidemics would either not be observed or specific known infectious agents would be identified by careful epidemiologic study as being associated with the epidemics. In fact, there has been an absence of association of KD with known infectious agents during epidemics and outbreaks despite careful study by epidemiologists in Japan, in the US at the Centers for Disease Control, and in other nations (5, 12, 13, 25). If any of multiple agents can cause KD, a substantially higher recurrence rate than the observed 1–3% in the US and Japan would be likely. Because the vast majority of patients do not develop other health problems following KD, an immune defect seems highly unlikely.

**Figure 1:**
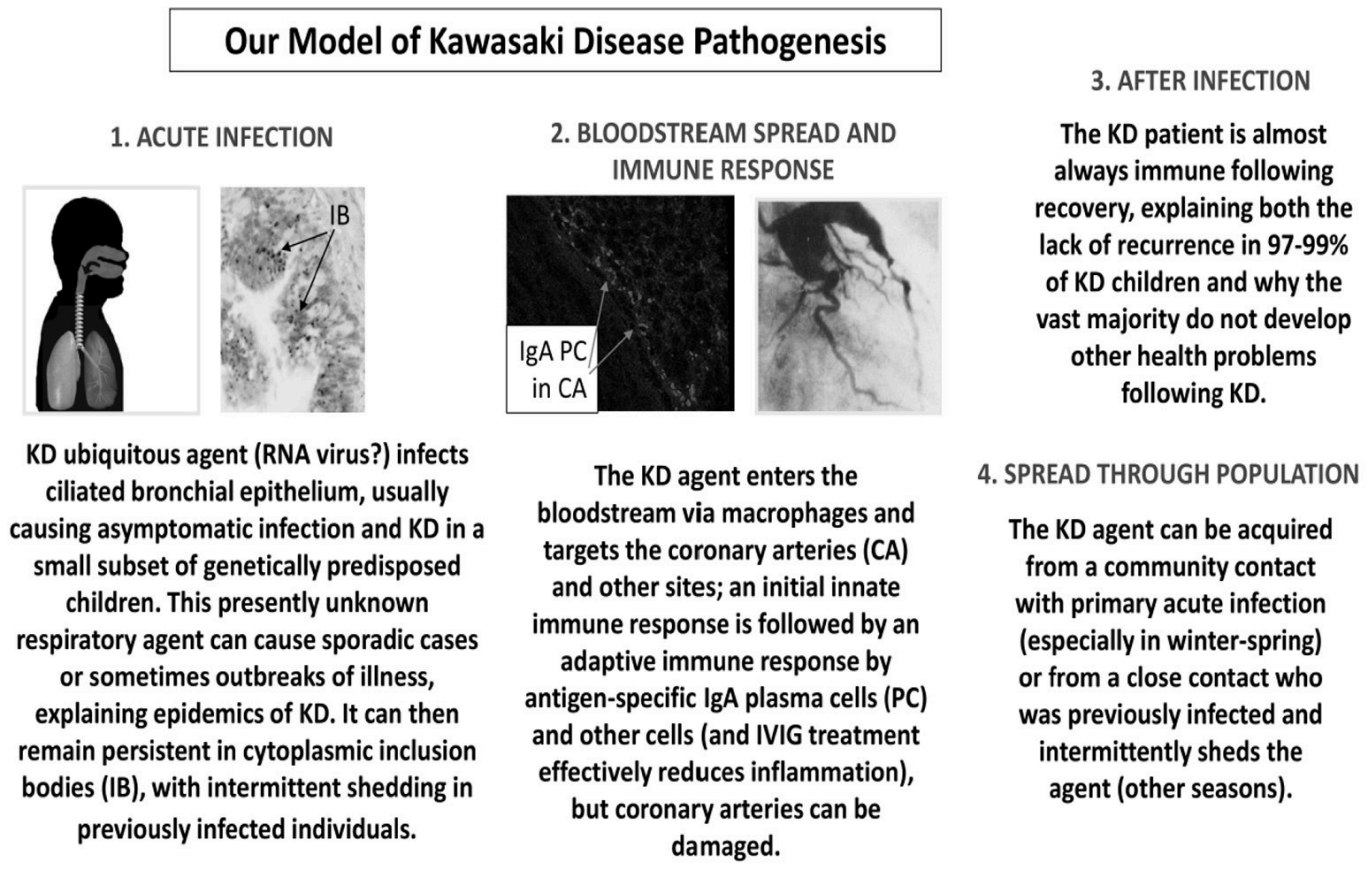
A proposed model of KD pathogenesis.

Our studies demonstrating an antigen-driven IgA immune response in acute KD and the presence of KD antigen in intracytoplasmic inclusion bodies in KD bronchial epithelium lead us to put forth the following model of KD pathogenesis (Figure 1). We propose that a presently unidentified (likely “new”) RNA virus infects ciliated bronchial epithelium, causing asymptomatic infection in most individuals and KD in a small subset of genetically predisposed children. Children < 6 months of age are less susceptible because of passive maternal antibody. The virus can result in sporadic cases of KD or in outbreaks. The agent can remain persistent in cytoplasmic inclusion bodies, with intermittent shedding into the respiratory tract of previously infected individuals. It can enter the bloodstream via macrophages and target particularly the coronary arteries and also other sites. Antigen-specific IgA plasma cells (17, 19, 20, 22, 23) and CD8 T cells (14, 15, 26) respond to the infection, but coronary arteries can be damaged. The provision of specific antibodies directed at the ubiquitous KD agent could explain the efficacy of intravenous gammaglobulin (IVIG) in the treatment of KD. These specific antibodies are present in IVIG because most adult donors were asymptomatically infected during young childhood, which accounts for the reduced prevalence in older children and the rarity of KD in adults. After infection, 97–99% of KD patients are immune to the agent and do not have a recurrence of KD. The agent can be spread through the population either from community contacts with asymptomatic primary infection particularly in the winter-spring, or from a close contact who had been previously infected and then intermittently sheds the agent, resulting in cases during other seasons. We believe that our model, although speculative, fits clinical and epidemiologic findings in KD much better than other currently proposed speculative models.

Identification of the etiology of KD is the most important research goal in the field. With this information, a diagnostic test can be developed, therapy improved, and prevention become possible. Hopefully, in the near future, the etiology can be discovered using synthetic antibodies derived from KD patients' B cell immune response to the triggering agent.

## Author Contributions

AR and SS contributed equally to conceiving the topics covered and in authoring the work.

### Conflict of Interest Statement

The authors declare that the research was conducted in the absence of any commercial or financial relationships that could be construed as a potential conflict of interest.
